# Extended Perioperative Antibiotic Coverage in Conjunction with Intraoperative Bile Cultures Decreases Infectious Complications after Pancreaticoduodenectomy

**DOI:** 10.1155/2016/3031749

**Published:** 2016-04-11

**Authors:** Amir H. Fathi, Terence Jackson, Mehdi Barati, Babak Eghbalieh, Kelly A. Siegel, Christopher T. Siegel

**Affiliations:** ^1^Department of Surgery, University of California San Francisco, Fresno Medical Education Program, Fresno, CA 93721, USA; ^2^Department of Surgery, University Hospitals Case Medical Center, Case Western Reserve University, Cleveland, OH 44106, USA; ^3^Economic Department, University of Wisconsin, Milwaukee, WI 53211, USA; ^4^Division of Hepatobiliary and Transplant Surgery, Department of Surgery, University Hospitals Case Medical Center, Case Western Reserve University, Cleveland, OH 44106, USA

## Abstract

*Background*. Bile contamination from the digestive tract is a well-known risk factor for postoperative complications. Despite the literature concerning prevalence of bacterobilia and fungobilia in patients with biliary pathologies, there are no specific recommendations for perioperative antimicrobial coverage for biliary/pancreatic procedures. We evaluated the effect of at least 72 hours of perioperative broad spectrum antibiotic coverage on outcomes of pancreaticoduodenectomy (PD).* Materials and Methods*. A retrospective review of all patients at Case Medical Center of Case Western Reserve University undergoing PD procedure, from 2006 to 2011, was performed (*n* = 122). Perioperative data including demographics, comorbidities, biliary instrumentation, antibiotic coverage, culture results, and postoperative outcomes were analyzed. Propensity score matching method was used to match the patients according to duration of antibiotic coverage into two groups: 72 hours (A72) and 24 hours (A24).* Results*. Longer broad spectrum antibiotic coverage in group A72 resulted in significantly less surgical site infections after PD, compared to routine 24 hours of perioperative antibiotics in group A24. This study did not reveal a statistically significant decrease in postoperative fungal infections in patients receiving preoperative antifungals.* Conclusion*. Prolonged perioperative antibiotic therapy in conjunction with intraoperative bile cultures decreases the short-term infectious complications of PD, with no significant increase in* Clostridium difficile* colitis incidence.

## 1. Introduction

In 1935, pancreaticoduodenectomy (PD) was formally described as a two-stage operation by Whipple et al. in Annals of Surgery [1]. Operative mortality from Whipple et al.'s original report to well into the 1970s was around 25% [2]. Since then, advances in surgical techniques and perioperative management have decreased the mortality associated with PD to current rates of less than 3–5%. Despite significant improvements in mortality, morbidity rates, even in high volume centers, are still substantial and affect up to 60% of patients [2–5]. Infectious complications account for more than 35% of these morbidities with sometimes deleterious consequences [3, 4]. To date, unlike other complications such as delayed gastric emptying or pancreatic fistula, infectious complications have been poorly studied [3, 4].

In 1999, Mangram and colleagues recommended the perioperative antimicrobial prophylaxis in the Center for Disease Control's guidelines [5]. Currently, the Surgical Care Improvement Project (SCIP) mandates every hospital to use perioperative antibiotics with proper timing in order to reduce perioperative complications [6, 7]. The deficiency of current routine antibiotic prophylaxis against biliary pathogens has been frequently confirmed by several high volume centers. Despite the literature concerning prevalence and trends of bacterobilia and fungobilia in patients with biliary/pancreatic pathologies, SCIP has yet to modify their recommendations for perioperative antibiotic coverage for PD [8–10].

In this study, we sought to evaluate the effect of longer duration broad spectrum antibiotic coverage and also perioperative antifungals on postoperative outcomes of PD. Evaluated outcomes included surgical site infections, bacteremia, intra-abdominal abscesses, anastomotic leaks, length of stay, rates of readmission, reoperation, and mortality. As shown in recent publications, we also hypothesize that routine utilization of intraoperative bile cultures with subsequent treatment of positive incubation results would reduce the infectious complications of PD.

## 2. Methods

A retrospective review of 122 patients at Case Medical Center of Case Western Reserve University undergoing PD, from 2006 to 2011, was performed. The approval for this study was obtained from the institutional review board (IRB).

These operations were performed by four senior surgeons with HPB or transplant surgery background. PD without pylorus preservation, including regional lymphadenectomy, was the operation of choice, as previously described [11]. Patients' demographic information including age, sex, and comorbidities of diabetes mellitus, chronic obstructive pulmonary disease, hypertension, hyperlipidemia, and also history of smoking and alcohol intake were obtained from medical records. Perioperative data including preoperative instrumentation history, operative details, cultures and sensitivities, and postoperative outcomes were extracted. The diagnosis of cancer was also recorded.

All postoperative complications, occurring within 30 days of the index operation, were documented, using standardized definitions. Pancreatic fistula was defined and graded by the definition proposed by International Study Group of Pancreatic Fistula (ISGPF) [12]. Hospital length of stay (LoS) was calculated from day of index surgery through and including the day of discharge.  Table 1 summarizes the postoperative events and their descriptions in this study.

Intraoperative characteristics records from the anesthesia documents were reviewed. The duration and choice of perioperative antibiotic regimen or antifungal coverage were mainly based on individual surgeon's practice. These regimens included piperacillin/tazobactam and 3rd generation cephalosporins, such as cefotetan or cefoxitin. In cases of penicillin allergy, intravenous ciprofloxacin and metronidazole were used as broad spectrum coverage. Antifungal coverage was achieved by administration of fluconazole intravenously. Intraoperative bile culture samples were sent for the majority of patients. All intraoperative cultures were collected as soon as the biliary tree or gallbladder was accessed. Based on the duration of perioperative broad spectrum antibiotic coverage, these 122 patients were divided into two main groups. The A72 group included 38 patients who received at least 72 hours of antibiotic coverage. In this group the antibiotics were started prior to incision and continued until the intraoperative cultures were incubated for 72 hours. At day 3, when the culture results were available, the antibiotics were either discontinued or tailored for positive cultures and continued for at least 10 days. The A24 group had 74 patients, receiving only 24 hours of perioperative antibiotics. All of the A24 patients received a single preoperative dose prior to incision. Intraoperative cultures were collected for 36 patients in the A72 group and for 15 patients in the A24 group.

### 2.1. Propensity Score Matching

In order to eliminate the selection bias, patients in the A24 and A72 groups were matched using the propensity score method. The propensity score for an individual patient was calculated by multivariate logistic regression model. The covariates used include age, sex, comorbidity, preoperative instrumentation of biliary system, and diagnosis of pancreatic cancer. Patients in A72 and A24 groups were one-to-one matched by the closest propensity score without replacement, using STATA software ver. 13 (StataCorp LP). This method comprises randomly ordering A72 and A24 patients, then selecting the first A72 subject, and finding the first A24 patient with the closest propensity score. As a result, we compared 37 patients from the A72 group with 37 matched A24 patients. One patient from A72 group was eliminated by software as a propensity score outlier.

### 2.2. Statistical Analysis

Baseline characteristics and postoperative outcomes of study patients were compared using a paired *t*-test for continuous variables and Chi-square or Fisher's exact test for categorical ones. The same statistical software was utilized to analyze the data set. In the two-tailed test, statistical significance was set at *P* < 0.050.

## 3. Results

Clinicopathological characteristics of study patients, before and after propensity score matching, are presented in Tables 2 and 3. The covariates including age, sex, comorbidity (hypertension, diabetes mellitus, chronic obstructive pulmonary disease, hyperlipidemia, and also history of smoking and alcohol intake), diagnosis of cancer, and history of preoperative instrumentation were considered for propensity score matching. The diagnosis of cancer was significantly different between two groups. The cancer patients received more antibiotics than noncancerous patients, which could be explained with the overall higher rate of instrumentation in these patients.

### 3.1. Effect of Preoperative Instrumentation

In order to factor in the effect of preoperative instrumentation into the postoperative complications, we compared the instrumentation rates between two groups. Patients in group A72 underwent significantly higher preoperative instrumentation (with or without propensity score matching) than those in group A24 (83% versus 59%, *P* = 0.038). Despite higher preoperative instrumentation, patients receiving 72 hours of antibiotics had significantly lower surgical site infections (*P* = 0.047) compared to those who received 24 hours of antibiotics. Rates of bacteremia and intra-abdominal deep organ/space infections were not significantly different between the two groups.

### 3.2. Postoperative Outcomes

Table 4 summarizes the postoperative outcomes after propensity score matching. Postoperative infectious complications were found in 51% of A72 group compared to 70% in A24 group. There was no difference in intra-abdominal abscess formation (*P* = 0.75), anastomotic leak (*P* = 0.08), pancreatic fistula formation rate (*P* = 0.11), or bacteremia (*P* = 1.0) between the two groups. However, longer duration of antibiotic treatment was associated with a significant decrease in postoperative surgical site infections (*P* = 0.04). Interestingly, prolonged use of perioperative antibiotics in A72 group was not associated with an increase in postoperative* Clostridium difficile* colitis incidence (*P* = 0.67).

This study did not reveal a significant difference in readmission rate (*P* = 0.62), reoperation rate (*P* = 0.79), or hospital stay duration (*P* = 0.88) between two groups. Mean length of stay for A72 group was 10.13 days, compared to 11.08 days for A24 group. There was one case of mortality in each group.

### 3.3. Culture Analysis

Intraoperative and postoperative culture results, in conjunction with clinically significant infections (Table 1) in the A72 and A24 groups, were analyzed in detail.

During postoperative care, necessary cultures were sent in both groups when clinically indicated from blood, surgical sites, drains, and intra-abdominal percutaneous aspirations.

These pathogens included fungi (*Candida albicans*,* Candida glabrata*,* Candida krusei*, and* Candida parapsilosis*), Gram negative bacilli (*Enterobacter*,* Pseudomonas*,* Klebsiella*, and* Escherichia*), anaerobes (*Enterococcus*,* Peptostreptococcus*), and coagulase positive and coagulase negative staphylococci.

*Klebsiella* (18.1%) and* Enterococcus* (13.6%) were the most common pathogens isolated from intraoperative bile cultures.* Enterococcus* species were the most common pathogens grown from surgical sites (27.3%) as well as intra-abdominal infections (25%). The total number of positive, intraoperative cultures was not significantly different between the A72 and A24 groups. Interestingly, in patients with positive postoperative cultures, there was 25% concordance with positive intraoperative cultures. In other words, 25% of patients grew the same organism from both intraoperative and postoperative culture samples.

This study did not reveal a statistically significant decrease in postoperative fungal infections in patients receiving preoperative antifungals.

## 4. Discussion

Despite the literature concerning the incidence and prevalence of bacterobilia and fungobilia in patients undergoing biliary/pancreatic procedures such as PD, there is no consensus for the specifics and duration of antibiotic prophylaxis. Bile serves as an optimal substrate for many bacteria and fungi, particularly when the biliary tract has been instrumented [13, 14]. Preoperative biliary stenting has been associated with a significant increased risk for various postoperative complications, including wound infections and intra-abdominal abscesses [15–17]. In concordance with existing literature, in our study, preoperative instrumentation was strongly associated with high incidence of positive intraoperative cultures. Recently, medical economics has entered an era where reimbursement for medical services is highly dependent on certain “preventable” complications such as postoperative infections. Therefore, initiatives such as the National Surgical Quality Improvement Program (NSQIP) and the Surgical Care Improvement Project (SCIP) have focused on controlling factors associated with perioperative infections in an effort to reduce additional health care costs while improving patient outcomes. In a study on infectious complications following pancreatic resection, Kent et al. stressed that infection control is a critical surrogate indicator of quality [18].

The idea of prolonging the perioperative antibiotics to 72 hours is not novel. The American Society of Health-System Pharmacists (ASHP) suggests continuation of antibiotic prophylaxis for cardiothoracic surgery for up to 72 hours [7]. As such, the precedent for specializing perioperative antibiotics in subspecialty practice has been set. Bratzler et al. reported that the duration of intravenous antibiotic use was over 24 hours in 59.3% of patients undergoing major surgery in the United States [19]. Japanese surgeons, who use 72 hours of perioperative antibiotics, believe that surgical stress might deteriorate the host immune system, increasing the risk of postoperative complications, including surgical site infections [20].

In this study, perioperative data including patient demographics, preoperative instrumentation, operative details, cultures and sensitivities, and postoperative outcomes were extracted from electronic medical records and anesthesia documents. The mean length of postoperative follow-up was 14 months, ranging from 1 to 62 months. Despite significantly higher preoperative instrumentation rate in the A72 group, a statistically significant, lower incidence of surgical site infections was found in the 72 hours' antibiotic coverage group. The rates of bacteremia, intra-abdominal deep organ/space infections, leak, pancreatic fistula, or mortality were not significantly different. There was no significant difference in readmission or reoperation rates between the two groups. Furthermore, culture data analysis revealed 25% concordance between positive postoperative and intraoperative cultures results. In other words, 25% of patients grew the same organism from both intraoperative and postoperative culture samples.

Two recent studies evaluated the effect of extended antibiotic therapy on infectious complications following PD [3, 4]. They both concluded that prolonged broad spectrum antimicrobial therapy will decrease the overall rates of postoperative infectious complications. Based on our findings and the data from mentioned papers, we suggest that preoperative broad spectrum antibiotics in conjunction with routine use of intraoperative bile cultures would improve the postoperative outcomes of PD. We propose to continue the antimicrobial regimen until the intraoperative culture results return. At our institution, culture results returned in about 72 hours. Thereafter, we either discontinued antibiotics or tailored them according to culture findings for a duration of at least 10 days. The term “biliary candidiasis” is used to describe the presence of yeast in otherwise sterile bile. Reports of biliary candidiasis over the last few years have increased interest in its prevalence and clinical significance [13]. The use of prophylactic antifungals, however, has been largely confined to high risk surgical patients in critical care units and immunocompromised patients. Studies have shown the efficacy of antifungals in preventing fungal infections particularly intra-abdominal invasive candidiasis and candidemia, in high risk surgical patients like those with recurrent gastrointestinal perforation and anastomotic leakage or acute necrotizing pancreatitis [21–24]. Mohammed et al. were the first group who investigated the incidence of fungal organisms detected by intraoperative bile culture in patients undergoing PD. They reported one wound infection and two intra-abdominal abscesses positive for fungus. They concluded that significance of biliary candidiasis and the value of its treatment are more controversial than the concept of treating bacteriobilia [4]. Our study did not reveal a statistically significant decrease in postoperative fungal infections in patients receiving preoperative antifungals. These infectious complications included one surgical site infection and nine deep organ/space abscesses growing different* Candida* species.

Inappropriate use of antibiotics has been associated with an increased incidence of colitis,* Clostridium difficile*-associated disease, and the development of antibiotic resistance [25]. In this study we did not encounter any apparent increase in the incidence of antibiotic-associated colitis with extended antibiotic treatments.

Considering the retrospective nature of the study and relatively small sample size, the length of perioperative antibiotics and inclusion of antifungals may not be the sole determinant of the decrease in incidence of surgical site infections. Also, the quality of pancreas texture was not consistently mentioned in all of the operative notes. Therefore, we used the cancer diagnosis as a surrogate for pancreatic tissue consistency. It was matched between two groups with propensity score matching system to eliminate the effect of pancreas softness on the fistula and leak outcomes. To the authors' knowledge, there have been only two recent retrospective studies, evaluating the extended antimicrobial therapy. In concordance with our findings, they also concluded that prolonged antibiotic therapy would decrease the short-term postoperative complications. This retrospective study adds to the foundation upon which future randomized prospective studies could further investigate the role of extended antimicrobial therapy on postoperative infectious complications, to generate uniform guidelines.

## Figures and Tables

**Table 1:** Postoperative events and their description.

Bacteremia	Blood culture proven bacteremia in a patient within 30 days of surgery.

Wound infection/dehiscence	Both superficial and deep surgical site infections, and/or wound dehiscence, diagnosed by laboratory confirmation or by attending physician within 30 days of surgery.

Organ/space infections	Intra-abdominal/solid organ intraparenchymal fluid collection, with purulent drainage from a drain or radiographic evidence with FNA sampling all confirmed by laboratory, presenting within 30 days of surgery.

Readmissions	Any in-patient admission to the same or different hospital for adverse events related to PD, within 30 days of surgery.

Reoperation	Procedures under general, local, spinal, or epidural anesthesia including wound debridement, in-patient I&D, IR drainage, or outpatient surgery for adverse events related to PD within 30 days of surgery.

Anastomotic leak	Any fluid collection or drain output with amylase content 3x the serum level or total bilirubin > 5 mg/dL above the patient's serum bilirubin was considered a leak, regardless of daily output.

**Table 2:** Clinicopathological features of the study patients prior to propensity.

Clinicopathological feature	A24 (*n* = 84)	A72 (*n* = 38)	*P* value
Age, mean, y ± SD	69.09 ± 11.3	66.73 ± 11.64	0.279
Gender			0.195
Male	38 (69)	22 (57)	
Female	46 (31)	16 (43)	
Hx of preoperative instrumentation	49 (58)	31 (81)	0.009
Comorbidity	57 (67)	8 (78)	0.210
HTN	41 (48)	15 (39)	0.338
DM	19 (22)	13 (34)	0.178
COPD	1 (1)	4 (10)	0.032
HLD	19 (22)	7 (18)	0.60
Smoker	13 (15)	8 (21)	0.450
EtOH use	7 (8.3)	8 (21)	0.05
Diagnosis of cancer	63 (75)	36 (94)	0.007

**Table 3:** Clinicopathological features of the matched patients using propensity score.

Clinicopathological feature	A24 (*n* = 37)	A72 (*n* = 37)	*P* value
Age, mean, y ± SD	70.48 ± 10.44	67.02 ± 11.67	0.231
Gender			0.815
Male	20 (54)	21 (56)	
Female	17 (46)	16 (44)	
Hx of preoperative instrumentation	22 (59)	31 (83)	0.038
Comorbidity	22 (59)	29 (78)	0.130
HTN	16 (43)	14 (37)	0.636
DM	5 (13)	13 (35)	0.056
COPD	1 (2)	4 (10)	0.358
HLD	6 (16)	7 (18)	0.760
Smoker	6 (16)	8 (21)	0.553
EtOH use	4 (10)	8 (21)	0.345
Diagnosis of cancer	25 (67)	35 (94)	0.006

**Table 4:** Postoperative outcomes of matched patients.

Postoperative outcomes	A24 (*n* = 37)	A72 (*n* = 37)	*P* value
Infectious complication	26 (70)	19 (51)	0.096
Surgical site infection	6 (16)	1 (2.7)	0.047
Intra-abdominal abscess	7 (18)	5 (13)	0.754
Anastomotic leak	5 (13)	1 (2.7)	0.08
*C. difficile* infection	2 (5)	4 (10)	0.674
Bacteremia	3 (8.3)	2 (5.4)	1.000
Mortality	1 (27)	1 (2.7)	1.000
Readmission	14 (37)	12 (32)	0.626
Reoperation	11 (29)	9 (24)	0.794
Length of stay, mean, Day ± SD	11.08 ± 6.22	10.13 ± 5.26	0.882
Pancreatic fistula			0.11
Grade B	10	2	
Grade C	3	0	

## References

[1] Whipple A. O., Parsons W. B., Mullins C. R. (1935). Treatment of carcinoma of the ampulla of Vater. *Annals of Surgery*.

[2] Schmidt C. M., Powell E. S., Yiannoutsos C. T. (2004). Pancreaticoduodenectomy: a 20-year experience in 516 patients. *Archives of Surgery*.

[3] Sourrouille I., Gaujoux S., Lacave G. (2013). Five days of postoperative antimicrobial therapy decreases infectious complications following pancreaticoduodenectomy in patients at risk for bile contamination. *HPB*.

[4] Mohammed S., Evans C., Vanburen G. (2014). Treatment of bacteriobilia decreases wound infection rates after pancreaticoduodenectomy. *HPB*.

[5] Mangram A. J., Horan T. C., Pearson M. L. (1999). Guideline for prevention of surgical site infection, Hospital Infection Control Practices Advisory Committee. *Infection Control & Hospital Epidemiology*.

[6] Stulberg J. J., Delaney C. P., Neuhauser D. V., Aron D. C., Fu P., Koroukian S. M. (2010). Adherence to surgical care improvement project measures and the association with postoperative infections. *The Journal of the American Medical Association*.

[7] (1999). ASHP therapeutic Guidelines on antimicrobial prophylaxis in surgery. American Society of Health-System Pharmacists. *American Journal of Health-System Pharmacy*.

[8] Mezhir J. J., Brennan M. F., Baser R. E. (2009). A matched case-control study of preoperative biliary drainage in patients with pancreatic adenocarcinoma: routine drainage is not justified. *Journal of Gastrointestinal Surgery*.

[9] Pisters P. W. T., Hudec W. A., Hess K. R. (2001). Effect of preoperative biliary decompression on pancreaticoduodenectomy-associated morbidity in 300 consecutive patients. *Annals of Surgery*.

[10] Sohn T. A., Yeo C. J., Cameron J. L. (2000). Do preoperative biliary stents increase post-pancreaticoduodenectomy complications?. *Journal of Gastrointestinal Surgery*.

[11] Evans D. B., Christians K. K., Foley W. D., Fischer J. L. (2010). Pancreaticoduodenectomy and total pancreatectomy for cancer. *Mastery of Surgery*.

[12] Bassi C., Dervenis C., Butturini G. (2005). Postoperative pancreatic fistula: an international study group (ISGPF) definition. *Surgery*.

[13] Lenz P., Conrad B., Kucharzik T. (2009). Prevalence, associations, and trends of biliary-tract candidiasis: a prospective observational study. *Gastrointestinal Endoscopy*.

[14] Jethwa P., Breuning E., Bhati C., Buckles J., Mirza D., Bramhall S. (2007). The microbiological impact of pre-operative biliary drainage on patients undergoing hepato-biliary-pancreatic (HPB) surgery. *Alimentary Pharmacology and Therapeutics*.

[15] Heslin M. J., Brooks A. D., Hochwald S. N., Harrison L. E., Blumgart L. H., Brennan M. F. (1998). A preoperative biliary stent is associated with increased complications after pancreatoduodenectomy. *Archives of Surgery*.

[16] van der Gaag N. A., Rauws E. A. J., van Eijck C. H. J. (2010). Preoperative biliary drainage for cancer of the head of the pancreas. *The New England Journal of Medicine*.

[17] Tol J. A. M. G., Busch O. R. C., van der Gaag N. A., van Gulik T. M., Gouma D. J. (2012). The quandary of preresection biliary drainage for pancreatic cancer. *Cancer Journal*.

[18] Kent T. S., Sachs T. E., Callery M. P., Vollmer C. M. (2013). The burden of infection for elective pancreatic resections. *Surgery*.

[19] Bratzler D. W., Houck P. M., Richards C. (2005). Antimicrobial prophylaxis for surgery: an advisory statement from the National Surgical Infection Prevention Project. *Archives of Surgery*.

[20] Haga N., Ishida H., Ishiguro T. (2012). A prospective randomized study to assess the optimal duration of intravenous antimicrobial prophylaxis in elective gastric cancer surgery. *International Surgery*.

[21] Meijer W. S., Schmitz P. I. M., Jeekel J. (1990). Meta-analysis of randomized, controlled clinical trials of antibiotic prophylaxis in biliary tract surgery. *British Journal of Surgery*.

[22] Vardakas K. Z., Samonis G., Michalopoulos A., Soteriades E. S., Falagas M. E. (2006). Antifungal prophylaxis with azoles in high-risk, surgical intensive care unit patients: a meta-analysis of randomized, placebo-controlled trials. *Critical Care Medicine*.

[23] Eggimann P., Francioli P., Bille J. (1999). Fluconazole prophylaxis prevents intra-abdominal candidiasis in high-risk surgical patients. *Critical Care Medicine*.

[24] Senn L., Eggimann P., Ksontini R. (2009). Caspofungin for prevention of intra-abdominal candidiasis in high-risk surgical patients. *Intensive Care Medicine*.

[25] Muto C. A., Pokrywka M., Shutt K. (2005). A large outbreak of *Clostridium difficile*-associated disease with an unexpected proportion of deaths and colectomies at a teaching hospital following increased fluoroquinolone use. *Infection Control and Hospital Epidemiology*.

